# IL-10 Dependent Suppression of Type 1, Type 2 and Type 17 Cytokines in Active Pulmonary Tuberculosis

**DOI:** 10.1371/journal.pone.0059572

**Published:** 2013-03-27

**Authors:** Nathella Pavan Kumar, Venugopal Gopinath, Rathinam Sridhar, Luke E. Hanna, Vaithilingam V. Banurekha, Mohideen S. Jawahar, Thomas B. Nutman, Subash Babu

**Affiliations:** 1 National Institutes of Health—International Center for Excellence in Research, Chennai, India; 2 National Institute for Research in Tuberculosis, Chennai, India; 3 Government Stanley Medical Hospital, Chennai, India; 4 Laboratory of Parasitic Diseases, National Institutes of Allergy and Infectious Diseases, National Institutes of Health, Bethesda, Maryland, United States of America; National Institute for Infectious Diseases (L. Spallanzani), Italy

## Abstract

**Background:**

Although Type 1 cytokine responses are considered protective in pulmonary tuberculosis (PTB), their role as well as those of Type 2, 17 and immunoregulatory cytokines in tuberculous lymphadenitis (TBL) and latent tuberculosis (LTB) have not been well studied.

**Aim and Methods:**

To identify cytokine responses associated with pulmonary tuberculosis (TB), TB lymphadenitits and latent TB, we examined mycobacterial antigen-specific immune responses of PTB, TBL and LTB individuals. More specifically, we examined ESAT-6 and CFP-10 induced Type 1, Type 2 and Type 17 cytokine production and their regulation using multiplex ELISA.

**Results:**

PTB individuals exhibited a significantly lower baseline as well as antigen-specific production of Type 1 (IFNγ, TNFα and IL-2); Type 2 (IL-4) and Type 17 (IL-17A and IL-17F) cytokines in comparison to both TBL and LTB individuals. TBL individuals exhibited significantly lower antigen-specific IFNγ responses alone in comparison to LTB individuals. Although, IL-10 levels were not significantly higher, neutralization of IL-10 during antigen stimulation resulted in significantly enhanced production of IFNγ, IL-4 and IL-17A in PTB individuals, indicating that IL-10 mediates (at least partially) the suppression of cytokine responses in PTB.

**Conclusion:**

Pulmonary TB is characterized by an IL-10 dependent antigen-specific suppression of Type 1, Type 2 and Type 17 cytokines, reflecting an important association of these cytokines in the pathogenesis of active TB.

## Introduction

Exposure to *Mycobacterium tuberculosis* (Mtb) can result in a variety of outcomes, including the absence of any clinical or laboratory evidence of infection, latent infection without active disease, active pulmonary disease or active extra-pulmonary disease [1]. Although 2 billion people worldwide are infected with Mtb, only 5–10% of these individuals develop active disease, and the mechanism by which most individuals resist development of active disease is still not clear [1]. Amongst those who develop active disease, only a small proportion develop extrapulmonary disease and why extrapulmonary dissemination following initial infection occurs is also not known [2]. A wide range of specific and non-specific host immune responses are thought to contribute to the differential outcomes of infection and disease, although there is no unifying hypothesis to explain the differences seen.

The most well studied of the protective immune responses are T cell mediated responses, known to be central in the host control of Mtb infection [3]. The ability of CD4^+^ T cells to produce Type 1 cytokines (especially INFγ), that can activate phagocytes to contain/constrain the intracellular mycobacterial pathogen, is crucial in host protection [3]. The importance of IFNγ and IL-12 in protection against disease was evident from the increased risk of tuberculosis in individuals with deficiencies in either the IFNγ or IL-12 signaling pathways [4]. Similarly, other Type 1 cytokines such as TNFα are also important in protection by contributing to the establishment and maintenance of the granuloma, a well organized collection of innate and adaptive cells that forms [5]. CD4^+^ T cell subsets other than the Th1-type may also play a role in protection from tuberculous disease most notably the IL-17 producing CD4^+^ T cells (Th17 cells) that have been shown to mediate the recruitment of protective Th1 cells to the lung upon Mtb challenge in animals [6]. In contrast, a number of counterbalancing regulatory factors including regulatory T cells, IL-10 and TGFβ have been implicated in establishment of chronic Mtb infection, felt to reflect the down modulation of protective immune responses [7], [8]. In addition, the presence of antigen – specific CD4^+^ Th1 cells in the absence of active disease is considered to define latent infection [9], often defined by either the tuberculin skin test or the IFNγ release assays [10]. Therefore, latent infection is thought to reflect a critical balance between Th1 and Th17 responses that serve to control the pathogen and Th2 cells, regulatory T cells and immunoregulatory cytokines (e.g.,IL-10 and TGFβ) that limit immune-mediated pathology [9]. Apart from latent infection, a common form of less severe TB disease is TB lymphadenitis [2], a form thought to be associated with extra-pulmonary spread through a hematogenous or a lymphatic route.

To study roles of T cell cytokines and potential regulatory factors, we examined Mtb antigen-specific induction of Type 1, 2, and 17 responses as well as production of IL-10 and TGFβ in pulmonary TB (PTB), tuberculous lymphadenitis (TBL) and latent TB (LTB) individuals in an area highly endemic for tuberculosis. We observed that active pulmonary TB was characterized by a dimunition of spontaneous and antigen-specific production of Type 1, 2 and 17 cytokines. TBL individuals, in contrast to those with PTB, exhibited a reduction only in the production of Type 1 (but not Type-2 or -17) cytokines. The suppression of cytokine responses in PTB was primarily mediated by IL-10.

## Methods

### Study population

We studied a group of 71 individuals; 26 with PTB, 23 with TBL and 22 individuals with LTB (Table 1). Individuals with PTB were diagnosed by positive sputum acid-fast bacillus (AFB) Ziehl-Neelsen staining and solid cultures in Lowenstein - Jensen medium. Individuals with TBL were diagnosed on the basis of clinical examination and AFB staining and culture of fine-needle aspiration biopsies of lymph nodes. Individuals were diagnosed as having LTB on the basis of being positive in the Quantiferon-TB Gold in Tube (Cellestis) assay but having an absence of pulmonary symptoms concurrent with a normal chest radiograph. All subjects had been bacillus Calmette-Guérin (BCG) vaccinated at birth. All the individuals were HIV negative and blood was collected prior to commencement of anti-TB treatment. This clinical protocol was approved by the Institutional Review Board of the National Institute of Research in Tuberculosis, and informed written consent was obtained from all participants.

**Table 1:** Study population.

Study Demographics	PTB	TBL	LTB
No. of subjects recruited	26	23	22
No.of males	22	12	17
Median Age	38 (19–54)	33 (19–65)	30 (21–47)
Quantiferon Assay (Positive/Negative)	16/10		22/0

Hematology profile	PTB	TBL	LTB
Red Blood Cells (µl)	4.2 (3.3–5.7)	4.7 (3.3–5.7)	5.149 (4.3–5.9)
White Blood Cells (µl)	7.6 (3.6–12.8)	7.9 (5.1–13.1)	9.1 (5.8–15.6)
Lymphocytes (%)	18.35 (8.6–42.5)	25.66 (11–41.1)	31.9 (23.5–40.5)
Neutrophils (%)	65.04 (44.8–79.5)	56.74 (42.1–67.4)	56.4 (46.9–66.4)
Monocytes (%)	8.7 (5.7–19.6)	10.1 (5.3–14.2)	6.8 (4.9–9.8)
Eosinophils (%)	3.4 (1.1–9.3)	2.7 (0.7–7)	2.9 (1.5–5.9)
Basophils (%)	0.3 (0.1–1.5)	0.7 (0.5–2.5)	0.8 (0.4–1.2)
Platelets	320.4 (113–551)	314.5 (186–528)	315.6 (232–446)
Red Blood Cells (µl)	4.2 (3.3–5.7)	4.7 (3.3–5.7)	5.149 (4.3–5.9)

### Hematologic analysis

Hematology was performed on all patients using the Act-5 Diff hematology analyzer (Beckman Coulter). As shown in Table 1, there were no significant differences in the baseline hematological parameters between PTB, TBL and LTB individuals.

### Antigens

Mycobacterial antigens - PPD (Statens Serum Institute, Copenhagen, Denmark), ESAT-6 and CFP—10 (both recombinant proteins from Fitzgerald Industries Intl. Inc) were used as antigenic stimuli, and anti-CD3 antibody was used as positive control. Final concentrations were 10 µg/ml for PPD, ESAT-6 and CFP-10 and 5 µg/ml for anti-CD3.

### *In vitro* culture

Whole blood cell cultures were performed to determine the levels of cytokines. Briefly, whole blood was diluted 1∶1 with RPMI-1640 medium supplemented with penicillin/streptomycin (100 U/100 mg/ml), L-glutamine (2 mM), and HEPES (10 mM) (all from Invitrogen) and distributed in 12-well tissue culture plates (Costar, Corning Inc). The cultures were then stimulated with PPD, ESAT-6, CFP-10, or anti-CD3 or media alone. After 72 h, culture supernatants were collected and analyzed for cytokines. For cytokine neutralization experiments, whole blood was cultured in the presence of anti-IL-10 (5 µg/ml), anti-TGFβ (5 µg/ml) or isotype control antibody (5 µg/ml) (all from R& D Sytems) for 1 h following which PPD was added and cultured for a further 72 h.

### Enzyme-linked immunosorbent assay (ELISA)

Levels of cytokines in the culture supernatants were measured using Bioplex multiplex cytokine assay system (Bio-Rad). The cytokines analyzed were IL-2, IFNγ, TNFα, IL-4, IL-5, IL-10, IL-13 and IL-17A. IL-17F, IL-22 and TGFβ ELISAs were performed using the kit from R&D Systems.

### Statistical analysis

Geometric mean was used as the measure of central tendency. Comparisons were made using either the Kruskal-Wallis test with Dunn's multiple comparisons (unpaired comparisons) or the Wilcoxon signed rank test (paired comparisons). All statistics were performed using GraphPad Prism version 5 for Windows (GraphPad Software, Inc.).

## Results

### PTB is associated with lower levels of Type 1, 2, 17 and immunoregulatory cytokines produced spontaneously

**Figure 1:**
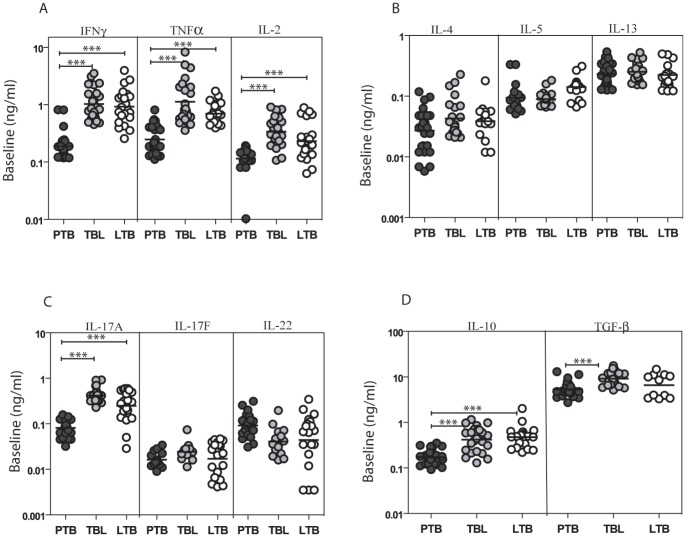
PTB is associated with decreased spontaneous production of Type 1, 2, 17 and immunoregulatory cytokines. Whole blood from PTB, TBL and LTB individuals was stimulated with media alone for 72 h, and levels of (A) Type 1 cytokines IFNγ, TNFα and IL-2; (B) Type 2 cytokines IL-4, IL-5, and IL-13; (C) Type 17 cytokines IL-17A, IL-17F and IL-22 and (D) Immunoregulatory cytokines IL-10 and TGFβ were measured by ELISA. Results are shown as scatterplots with each dot representing a single individuals. *P* values were calculated using the Kruskal-Wallis test with Dunn's multiple comparisons (* p<0.05, ** p<0.01, *** p<0.001).

To determine T cell cytokine profile in pulmonary, extra-pulmonary and latent TB, we measured levels of Type 1, 2, 17 and immunoregulatory cytokines following 72 h culture of whole blood from PTB, TBL or LTB individuals in the absence of antigen stimulation (Figure 1). As shown in Figures 1A and C, those with PTB exhibited significantly lower levels of spontaneously produced Type 1 [IFNγ: (geometric mean [GM] of 0.187 ng/ml in PTB; 1.03 ng/ml in TBL and 0.93 ng/ml in LTB), TNFα (GM of 0.246 in PTB; 1.13 in TBL and 0.697 in LTB), IL-2 (GM of 0.114 in PTB; 0.340 in TBL and 0.232 in LTB)] and Type 17 cytokines [IL-17A (GM of 0.080 in PTB; 0.417 in TBL and 0.248 in LTB) but not IL-17F or IL-22] in comparison to TBL and LTB individuals. No significant differences in Type 2 cytokines were observed among the three groups at baseline (Figure 1B). Finally, those with PTB individuals also exhibited significantly lower levels of the immunoregulatory cytokines – IL-10 (GM of 0.172 in PTB; 0.419 in TBL and 0.472 in LTB) and TGFβ (GM of 5.32 in PTB; 9.12 in TBL and 6.55 in LTB) in comparison to TBL and LTB individuals (Figure 1D). Thus, active PTB is characterized by lower levels of spontaneous production of T cell cytokines.

### PTB is associated with decreased production of antigen-specific Type 1 cytokines

**Figure 2:**
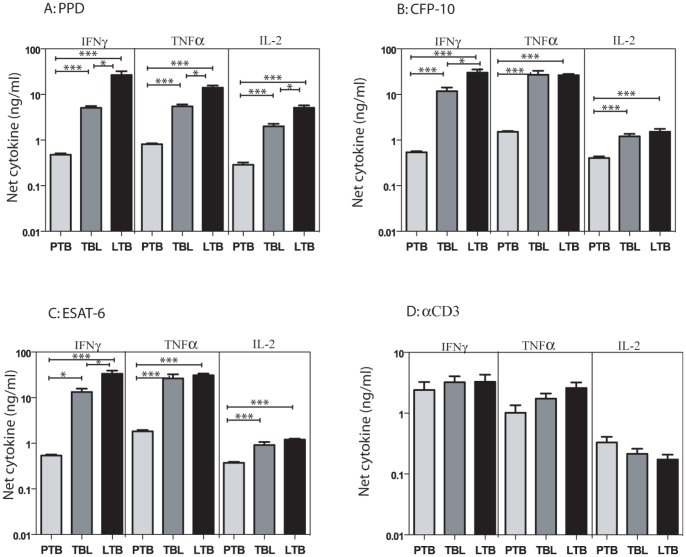
PTB is associated with decreased antigen-stimulated production of Type 1 cytokines. Whole blood from PTB, TBL and LTB individuals was stimulated with (A) PPD (10 µg/ml) or (B) ESAT-6 (10 µg/ml) or (C) CFP-10 (10 µg/ml) or (D) anti-CD3 (5 µg/ml) for 72 h, and levels of Type 1 cytokines IFNγ, TNFα and IL-2 were measured by ELISA. Results are shown as net cytokine production over media control. The bars represent geometric means and 95% confidence intervals. *P* values were calculated using the Kruskal-Wallis test with Dunn's multiple comparisons comparisons (* p<0.05, ** p<0.01, *** p<0.001).

To determine the impact of PTB, TBL or LTB on mycobacterial antigen-specific Type 1 cytokine responses, we measured levels of antigen – specific IFNγ, TNFα and IL-2. As shown in Figures 2A, B, C, PTB individuals exhibited significantly lower levels of IFNγ, TNFα and IL-2 in response to PPD [IFNγ: (geometric mean [GM] of 0.445 ng/ml in PTB; 4.20 ng/ml in TBL and 13.9 ng/ml in LTB), TNFα (GM of 0.796 in PTB; 4.41 in TBL and 11.2 in LTB), IL-2 (GM of 0.234 in PTB; 1.56 in TBL and 3.99 in LTB)]; ESAT-6 [IFNγ (GM of 0.519 in PTB; 7.96 in TBL and 23.6 in LTB), TNFα (GM of 1.73 in PTB; 12.7 in TBL and 28.9 in LTB), IL-2 (GM of 0.354 in PTB; 0.612 in TBL and 1.77 in LTB)]; CFP-10 [IFNγ (GM of 0.517 in PTB; 7.28 in TBL and 21.9 in LTB), TNFα (GM of 1.49 in PTB; 14.0 in TBL and 25.1 in LTB), IL-2 (GM of 0.372 in PTB; 0.918 in TBL and 1.32 in LTB)] but not anti-CD3 (Figure 2D) in comparison to both TBL and LTB individuals. Those with TBL exhibited significantly lower levels of IFNγ, TNFα and IL-2 in response to PPD and significantly lower levels of IFNγ only in response to ESAT-6 and CFP-10 in comparison to LTB individuals. Thus, active TB (both PTB and TBL) is characterized by adecreased mycobacterial antigen-specific Type 1 cytokine response.

### PTB is associated with decreased antigen-specific production of IL-4

**Figure 3:**
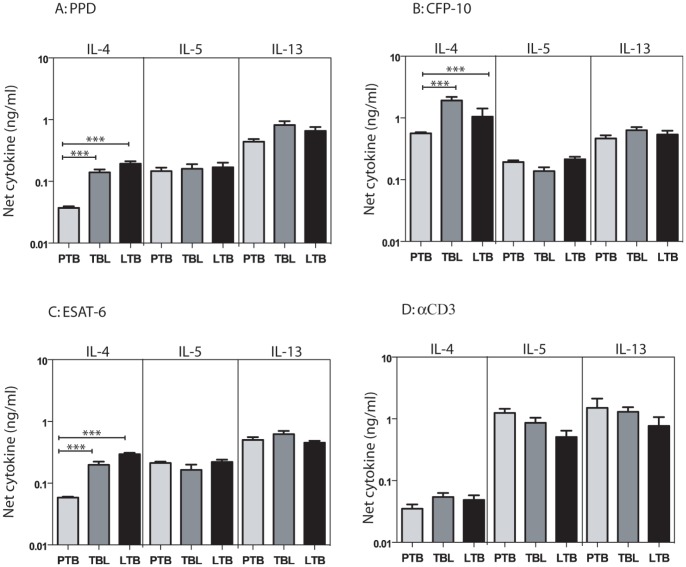
PTB is associated with decreased antigen-stimulated production of IL-4. Whole blood from PTB, TBL and LTB individuals was stimulated with (A) PPD (10 µg/ml) or (B) ESAT-6 (10 µg/ml) or (C) CFP-10 (10 µg/ml) or (D) anti-CD3 (5 µg/ml) for 72 h, and levels of Type 2 cytokines IL-4, IL-5 and IL-13 were measured by ELISA. Results are shown as net cytokine production over media control. The bars represent geometric means and 95% confidence intervals. *P* values were calculated using the Kruskal-Wallis test with Dunn's multiple comparisons comparisons (* p<0.05, ** p<0.01, *** p<0.001).

To determine the impact of PTB, TBL, or LTB on mycobacterial antigen-specific Type 2 cytokine responses, we measured antigen – specific levels of IL-4, IL-5 and IL-13. As shown in Figures 3A, B, C, PTB individuals exhibited significantly lower levels of IL-4 but not IL-5 or IL-13 in response to PPD (GM of 0.034 in PTB; 0.117 in TBL and 0.169 in LTB), ESAT-6 (GM of 0.057 in PTB; 0.160 in TBL and 0.285 in LTB), CFP-10 (GM of 0.549 in PTB; 1.37 in TBL and 0.868 in LTB) but not anti-CD3 (Figure 3D) in comparison to both TBL and LTB individuals. Those with TBL did not exhibit any significant differences in Type 2 cytokine production in comparison to LTB individuals. Thus, active pulmonary TB is characterized by a decreased antigen-specific IL-4 response.

### PTB is associated with decreased production of antigen-specific Type 17 cytokines

**Figure 4:**
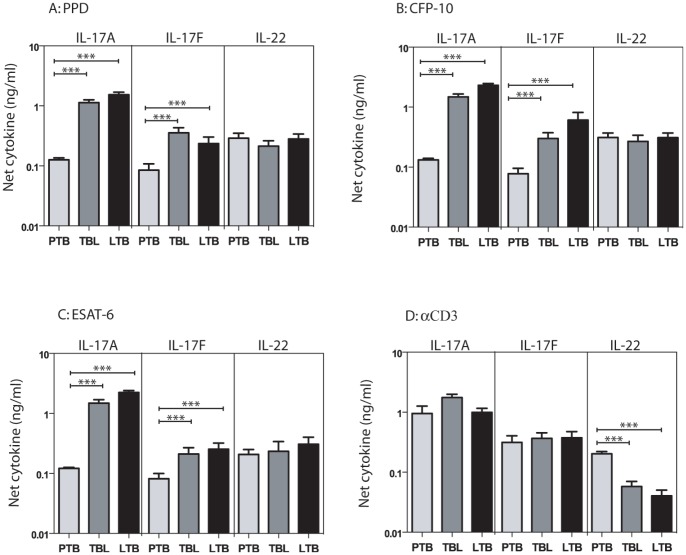
PTB is associated with decreased antigen-stimulated production of Type 17 cytokines. Whole blood from PTB, TBL and LTB individuals was stimulated with (A) PPD (10 µg/ml) or (B) ESAT-6 (10 µg/ml) or (C) CFP-10 (10 µg/ml) or (D) anti-CD3 (5 µg/ml) for 72 h, and levels of Type 17 cytokines IL-17A, IL-17F and IL-22 were measured by ELISA. Results are shown as net cytokine production over media control. The bars represent geometric means and 95% confidence intervals. *P* values were calculated using the Kruskal-Wallis test with Dunn's multiple comparisons comparisons (* p<0.05, ** p<0.01, *** p<0.001).

To determine the impact of PTB, TBL, or LTB on mycobacterial antigen-specific Type 17 cytokine responses, we measured antigen – specific levels of IL-17A, IL-17F and IL-22. As shown in Figures 4A, B, C, PTB individuals exhibited significantly lower levels of IL-17A and IL-17F in response to PPD [IL-17A (GM of 0.115 in PTB; 0.857 in TBL and 1.37 in LTB) and IL-17F (GM of 0.055 in PTB; 0.256 in TBL and 0.133 in LTB)], ESAT-6 [IL-17A (GM of 0.118 in PTB; 1.13 in TBL and 2.08 in LTB) and IL-17F (GM of 0.052 in PTB; 0.148 in TBL and 0.124 in LTB)], CFP-10 [IL-17A (GM of 0.126 in PTB; 1.20 in TBL and 2.15 in LTB) and IL-17F (GM of 0.050 in PTB; 0.185 in TBL and 0.228 in LTB)] but not anti-CD3 (Figure 4D) in comparison to both TBL and LTB individuals. Interestingly, antigen – induced IL-22 production was not significantly different between the 3 groups. Similarly, TBL individuals did not exhibit any significant differences in Type 17 cytokine production in comparison to LTB individuals. Thus, PTB (but not TBL) is characterized by a decreased antigen-specific Type 17 cytokine response.

### PTB is not associated with significant differences in the production of immunoregulatory cytokines

**Figure 5:**
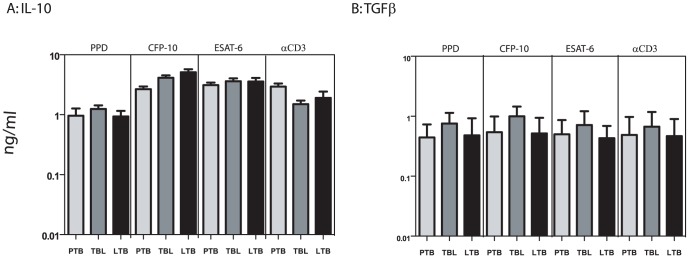
PTB is not associated with antigen – induced alterations in immunoregulatory cytokines. Whole blood from PTB, TBL and LTB individuals was stimulated with (A) PPD (10 µg/ml) or (B) ESAT-6 (10 µg/ml) or (C) CFP-10 (10 µg/ml) or (D) anti-CD3 (5 µg/ml) for 72 h, and levels of immunoregulatory cytokines IL-10 and TGFβ were measured by ELISA. Results are shown as net cytokine production over media control. The bars represent geometric means and 95% confidence intervals. *P* values were calculated using the Kruskal-Wallis test with Dunn's multiple comparisons comparisons (* p<0.05, ** p<0.01, *** p<0.001).

To determine the impact of active or latent infection or extra-pulmonary dissemination on mycobacterial antigen-specific immunoregulatory cytokine responses, we measured antigen – specific levels of IL-10 and TGFβ. As shown in Figure 5A, PTB individuals did not exhibit any significant difference in IL-10 production in response to PPD, ESAT-6, CFP-10 or anti-CD3 in comparison to both TBL and LTB individuals. Similarly, PTB individuals did not exhibit any significant difference in TGFβ production in response to PPD, ESAT-6, CFP-10 or anti-CD3 in comparison to TBL and LTB individuals (Figure 5B).

### Suppression of Type 1, 2 and 17 cytokines in PTB is overcome by IL-10 neutralization

**Figure 6:**
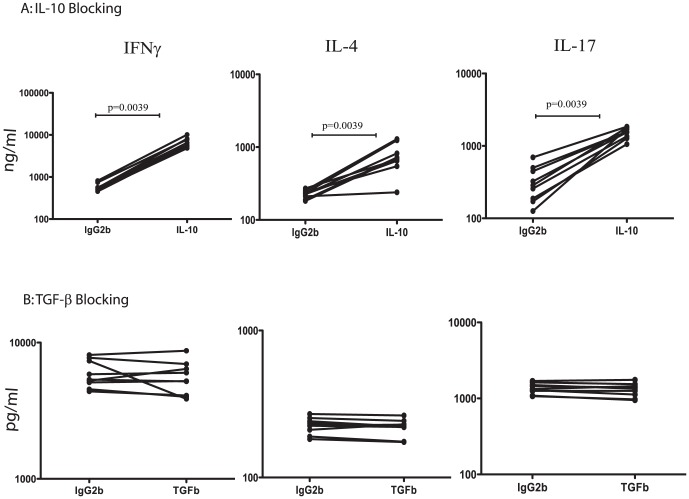
Neutralization of IL-10 but not TGFβ significantly enhances cytokine production in PTB. Whole blood from PTB individuals was stimulated with PPD (10 µg/ml) in the presence of anti-IL-10 Ab or anti-TGFβ Ab or isotype controls for 72 h and the levels of IFNγ, IL-4 and IL-17A were measured by ELISA. Results are shown as line graphs with each line representing a single PTB individual (n = 9). Results are shown as net cytokine production over media control. *P* values were calculated using the Wilcoxon signed rank test.

To determine the role of IL-10 and TGFβ in the suppression of antigen – specific T cell cytokine responses in PTB, we stimulated whole blood from PTB individuals with PPD in the presence of neutralizing antibodies for IL-10 or TGFβ or isotype controls for 72 h and measured levels of IFNγ, IL-4 and IL-17A. As shown in Figure 6A, neutralization or blockade of IL-10 resulted in significanly increased PPD-stimulated production of IFNγ (GM of 6.68 ng/ml with anti-IL-10 Ab vs. 0.592 ng/ml with isotype control), IL-4 (GM of 0.695 ng/ml vs. 0.224 ng/ml) and IL-17A (GM of 1.50 ng/ml vs. 0.291 ng/ml) in PTB individuals. Blockade of TGFβ had no effect on the PPD – induced production of IFNγ, IL-4 and IL-17A (Figure 6B). These data suggest that IL-10 plays a significant role in the suppression of the T cell response in PTB.

## Discussion

Infection with Mtb can produce a variety of outcomes, including active infection of the lungs—manifesting either as primary fulminant TB or as chronic, slowly progressing TB—, extra-pulmonary TB, which involves dissemination of the pathogen to sites other than the lung, and latent infection, which occurs when the initial infection is controlled but not completely eliminated [11]. The vast majority of individuals infected with Mtb mount an efficient cell-mediated immune response that leads to LTB with no clinical signs of disease [9]. However ∼5–10% of Mtb-infected individuals will progress to active disease [9]. Amongst those with active disease, a small percentage have extra-pulmonary manifestations of TB, including TB lymphadenitis (a clinically less severe form of disease) that accounts for 30–40% of extra-pulmonary infections [2]. Although a number of mechanisms have been described for the development of a protective immune response that restricts and controls infection and thus prevents progression to active disease, the reasons underlying active disease progression remain poorly understood.

Although CD4^+^ T cells have been shown to be of crucial importance in resistance to infection and/or disease [3], the role of the various CD4^+^ T cell subsets remains to be clarified, especially in extra-pulmonary forms of tuberculosis in an high – endemic setting. Hence, we have examined the pattern of Type 1, 2, 17 and immunoregulatory cytokine production with or without mycobacterial Ag stimulation in three groups of individuals – PTB, TBL and LTB. Th1 cells are essential for resistance to TB both in mice and in humans. Deficiencies in the IL-12-IFNγ-Stat1 pathway leads to disseminated mycobacterial infection in humans and to abrogation of resistance in mice [4], [12]. In addition, TNFα, another Th1 cytokine, is of almost equal importance, as treatment with biologics (e.g., anti-TNFα antibody) for inflammatory disorders such as rheumatoid arthritis has caused reactivation of TB in some individuals [13]. Our data reveal an impairment in the production of Type 1 cytokines both produced spontaneously and following Ag stimulation in PTB and in the production of Type 1 cytokines (especially IFNγ) following Ag stimulation in TBL individuals. The two important implications suggested by these results are that (1) Type 1 cytokine production is potentially a sensitive marker of resistance to infection and (2) IFNγ production is a possible mediator of protection against extra-pulmonary spread of TB. While the finding that Type 1 cytokines could potentially be involved in protection against active pulmonary disease is not novel, our finding that Type 1 cytokine production is compromised in both active PTB and TBL is quite interesting. Our data also imply that there exists a gradient in the production of Type 1 cytokines with the most severe disease (PTB) manifesting the lowest levels of cytokines (even at baseline) and most contained form of infection (LTB) exhibiting the highest production. However, it is also possible that the association between Type 1 cytokines and PTB and TBL is more a marker of pulmonary infection and/or extra-pulmonary dissemination rather than a correlate of protective immunity, since we have not established cause and effect in this study.

Increased Th2 responses have been typically postulated to play a role in susceptibility to TB, as IL-4 and IL-13 can undermine Th1-mediated immunity and drive inappropriate alternative activation of macrophages and inhibit autophagic control of Mtb [14], [15]. Our study reveals that neither spontaneously produced nor antigen – induced production of Type 2 cytokines is associated with disease development, since IL-4 levels were consistently and significantly lower in PTB individuals compared to TBL and LTB. Thus, Th2 responses are equally compromised in active pulmonary TB and these responses are modulated similar to Th1 responses by immunoregulatory mechanisms, especially IL-10.

We also examined induction of Type 17 responses in latent TB. Although Th17 cells are not as important as Th1 cells in mediating protection against primary Mtb infection, IL-17 appears to be critical in induction of Mtb-specific memory response and mediation of protection against challenge infections and during vaccinations [16], [17], [18], [19]. In addition, the IL-23/IL17 axis has been found to be important in human immune response to TB [20], [21], [22], [23]. In addition to IL-17A, we also examined the production of IL-17F and IL-22, two cytokines associated with Th17 responses but whose role in TB remains poorly explored. Our data clearly reveal an important relationship between the under-production of Type 17 cytokines and the presence of active pulmonary TB. Thus, both baseline and antigen –driven production of IL-17A and IL-17F are compromised in PTB but not in TBL. In addition, our data also show IL-22 is not associated with active TB as previously thought [23]. Finally, our data do not reveal any significant alterations in either spontaneously produced or antigen – driven levels of Type 2 or 17 cytokines in TBL compared to LTB. Therefore, it is highly likely that Type 2 and Type 17 cytokines have very little, if any, role to play in the pathogenesis of TBL and cytokine responses in this condition are essentially similar to another contained form of TB infection (ie LTB).

A characteristic feature of the host response to Mtb is the capacity to balance the immune response to restrict the growth of the pathogen while limiting the damage inflicted upon host tissues [7]. One of the mechanisms involved in limiting tissue damage mediated by pro-inflammatory responses is the production of IL-10 and TGFβ [7], [8]. IL-10 is known to inhibit host protective immune responses to certain pathogens [24], [25]. In experimental Mtb infection, IL-10 has been shown to inhibit host protective anti-microbial mechanisms and thereby enhance susceptibility to infection [8]. In human studies, elevated levels of IL-10 have been detected in the lungs and serum of those with active PTB [26], [27], [28] and neutralization of IL-10 has been shown to promote T cell proliferation and IFNγ production [29], [30], [31]. Based on these studies, we postulated that IL-10 could play an important role in modulation of cytokine responses in TB infection. However, we did not detect any significant increase in the antigen – driven levels of IL-10 in PTB or TBL individuals compared to LTB. While the reason behind the differences in IL-10 levels in our study from previous studies remain to be explored, it is likely to reflect differences in both host and pathogen factors, including host and pathogen genotypes and presence of co-existent infections. Nevertheless, neutralization of IL-10 prior to stimulation with Mtb antigen had a profound effect in overcoming the impaired cytokine production in PTB. To our knowledge, this study is the first to demonstrate a role for IL-10 in the suppression of Type 2 and Type 17 cytokine responses in tuberculosis. In addition, the absence of increased levels of antigen – stimulated IL-10 in TBL argues against a role for this cytokine in TBL, although this needs further exploration. Nevertheless, IL-10 is clearly an important regulatory mechanism in tuberculosis, with the ability to modulate the different arms of CD4+ T immunity.

Another mediator of immunsuppression in active TB is TGFβ. TGFβ has been shown to be produced at increased levels in active TB individuals compared to tuberculin skin test positive individuals in response to Mtb antigens. Moreover, defective T cell proliferation and cytokine production in active TB cases was shown to be dependent on TGFβ [32], [33], [34]. Our data, however, failed to reveal any significant difference in either the spontaneous production of TGFβ or in the capacity of TGFβ to modulate Type 1, 2 or 17 cytokines and therefore, suggest that TGFβ, unlike IL-10, plays only a minor role in the active suppression of cytokine responses in PTB in an endemic setting.

In summary, we have examined the modulation of host cytokines both in different forms of TB by comparing antigen – specific cytokine responses PTB, ETB and LTB individuals. Our study is limited by the fact that we examined only peripheral immune responses. Since data concerning lymphocyte recruitment or immunological responses at the site of infection – lungs in the case of PTB and lymph nodes in the case of TBL were not analyzed in our study, it is possible that our data reflect the compartmentalization of immune responses in TB pathogenesis. Thus, our findings in the periphery could also reflect preferential migration of Th1 and Th17 cells to the site of infection. Nevertheless, our study provides certain novel insights into the pathogenesis of pulmonary TB and extra-pulmonary TB, the latter clearly differing in pathogenesis from the former. Our data also argue that the protective immune response to *Mtb* disease may be attributed to the fine balance between proinflammatory and immunoregulatory mechanisms. IL-10 represents one such regulatory mechanism that *Mtb* likely exploits to establish a chronic infection and may therefore serve as an important target for the design of novel immune therapies.
